# Supplementary material for: Exploring the relationship between telehealth utilization and treatment burden among patients with chronic conditions: A cross-sectional study in Ontario, Canada
Source: PLOS Digit Health. 2024 Oct 15;3(10):e0000610. doi: 10.1371/journal.pdig.0000610 (PMC11478863; doi:10.1371/journal.pdig.0000610)
Supplement: S1 Appendix — (PDF) [file pdig.0000610.s001.pdf]

Appendix 1. Cross-sectional Study-Treatment burden questionnaire

Take a moment and consider everything you have to do to take care of your health. Please rate the burden or problem associated with each of the following items.

All questions are answered on a **0** (no burden) to **10** (high burden)

Questions	0<br><br>No Burden	1	2	3	4	5	6	7	8	9	10<br><br>High Burden
**How would you rate the problems related to:**											
**1.The taste, shape, or size** of your tablets and/or **the annoyances** caused by your injections (for example: pain, bleeding, bruising, or scars)?											
2. **The number of times** you should take your medication daily?											
3. **The efforts you make not to forget to take your medications** (for example: managing your treatment when you are away from home, preparing and using pillboxes…)											
4. **The necessary precautions when taking your medication** (for example: taking them at specific times of the day or for meals, not being able to do certain things after taking medications such as driving or lying down…)											
a. **Regarding your medical follow-up, how would rate the problems related to:**											
5. **Lab tests and other exams** (for example: blood tests or radiology): frequency, time spent and associated nuisances or inconveniences											
6. **Self-monitoring** (for example: taking your blood pressure or checking your blood sugar): frequency, time spent and associated nuisances or inconveniences											
7. **Doctor visits and other appointments:** frequency and time spent for these visits and difficulties finding healthcare providers											
8. The difficulties you could have in your **relationships with healthcare**											

**providers (**for example: feeling not listened to enough or not taken seriously)											
9. **Arranging medical appointment** (doctor visits, lab tests and other exams) and **reorganizing your schedule** around these appointments											
How would you rate:											
10. The **administrative burden** related to healthcare (for example: work you have to do for hospitalization, reimbursements and/or obtaining social services)?											
11. The **financial burden** associated with your healthcare (for example: out of pocket expenses or expenses not covered by insurance)?											
12. The burden related to **dietary changes** (for example: avoiding certain foods or alcohol, having to quit smoking…)?											
13. The burden related to **doctors' recommendations to practice physical activity** (for example: walking, jogging, swimming…)?											
14. How does your healthcare impact **your relationships with others** (for example: needing assistance in everyday life, being ashamed to take your medication…)?											
15. 'The need for medical healthcare on a regular basis **reminds me of my health problems'**											
