# Supplementary material for: Exploring the relationship between telehealth utilization and treatment burden among patients with chronic conditions: A cross-sectional study in Ontario, Canada
Source: PLOS Digit Health. 2024 Oct 15;3(10):e0000610. doi: 10.1371/journal.pdig.0000610 (PMC11478863; doi:10.1371/journal.pdig.0000610)
Supplement: S2 Appendix — (PDF) [file pdig.0000610.s002.pdf]

Appendix 2. Telehealth usage scale

(a) Have you used the internet, telephone, smartphone device to do any of the following things in **the last 12 months for yourself** (not for other family members) (select all that apply)?

- ☐ Communicating with (phone/text message) your primary care provider (e.g. family doctor, nurse, dietician etc.)
- ☐ Scheduling/re-scheduling your appointments with primary care clinic
- ☐ Tracking or monitoring symptoms using your smartphone, tablet or computers

(b) Please indicate how often you do each of the following activities using smartphones, computers or tablets to manage your chronic conditions.

Never	Once a month	Once a week	Several times a week	Once a day	Several times a day

1. Communicate (call/text message/email) with your primary care team

Never	Once a month	Once a week	Several times a week	Once a day	Several times a day

2. Schedule or reschedule your appointments with the primary care clinic

Never	Once a month	Once a week	Several times a week	Once a day	Several times a day

3. Track/monitor your chronic disease-related symptoms using your smartphone, tablet, or computers

Never	Once a month	Once a week	Several times a week	Once a day	Several times a day
