# Supplementary material for: Exploring the relationship between telehealth utilization and treatment burden among patients with chronic conditions: A cross-sectional study in Ontario, Canada
Source: PLOS Digit Health. 2024 Oct 15;3(10):e0000610. doi: 10.1371/journal.pdig.0000610 (PMC11478863; doi:10.1371/journal.pdig.0000610)
Supplement: Attachment — Submitted filename: Plos Digital health-reviewer comments_responses-FT_AS (1).docx [file pdig.0000610.s003.docx]

Dear Editors and reviewers,

We would like to thank you and the reviewers again for the thoughtful feedback, which has enabled us to improve our manuscript. We have addressed each comment from the reviewers below in a table format. The requested changes are typed in red within the manuscript document. Please let us know if you have any questions.

| Reviewer comments | Responses | Page and line numbers |
| --- | --- | --- |
| **Reviewer 1** | | |
| Abstract |  |  |
| **Comment 1:** Mean or median plus SD or range should be shown for age and 2 decimal points seems excessive. | Corrected | Page 2, line 53 |
| **Comment 2:** The results should show the key findings (associations) in numbers as well as narrative with appropriate statistical parameters. | Due to the word count limit in the abstract, we could not add additional parameters and information to the current abstract. |  |
| Introduction: |  |  |
| **Comment 3:** Please delineate positive versus negative factors (e.g. exercise vs troubleshooting technology) and those that are require patient involvement for their health (e.g. taking medications or exercise vs using technology or attending appointments) vs that contribute to a patients treatment burden. For instance, if telehealth causes the patient to exercise more hours at home but have to spend less time taking the bus to the clinic, is that an increase in burden? | Thanks for the important suggestion. We have addressed this in the introduction sections.  Moreover, there is a lack of research distinguishing between necessary burdens, such as exercise that positively contributes to patients' health, and avoidable burdens, such as administrative tasks associated with paperwork. Telehealth can help mitigate avoidable burdens by offering technologies such as online patient portals, which reduce the need for patients to complete paperwork for providers. Additionally, telehealth can support health-promoting activities by providing virtual self-management programs and classes. However, there is insufficient evidence on the overall impact of telehealth on treatment burden. | Page 5 lines 126-132 |
| **Comment 4:** Similarly, are the SES issues related to access or burden? Differentiate these. If a lower SES patient is less often offered video telehealth because they don’t have a phone or internet, is the telehealth a burden to them if they don’t use it? Limited “access” is not the same as “burden”. | Thanks for this suggestion. We agree a distinction between access and burden is significant. Hence, we have reorganized the paragraph to highlight why low SES patient may experience higher treatment burden due to technology use.  Clinicians are increasingly concerned about the potential treatment burden telehealth may pose, especially for patients with low socioeconomic status (SES). (25) This population is more likely to have low digital literacy, which could make telehealth use particularly burdensome for them. | Page 4, Line 111-114 |
| **Comment 5:** Would it be considered mitigating the burden to the patient of requiring exercise three times a week supported by telehealth if the patient simply chose not to exercise? This differential value needs to be considered. It would be less burden but less desirable/effective. | Addressed in comment 3. | Page 5 lines 126-132 |
| Methods: | | |
| **Comment 6:** **Study population** – please indicate if the CHC and social media recruited patients were similar or different given comments on CHS marginalized focus. Also, 13% of patients made >$100k. Describe. | We have addressed this comment in the limitation section:  Additionally, although we specifically recruited participants through CHCs to target patients with low SES, 13% of our study population had incomes over 100,000 CAD. Previous studies have highlighted the difficulty of recruiting low SES patients for research. Moreover, we cannot differentiate the characteristics of those recruited from CHCs versus social media, as the surveys were anonymous and this data was not collected. | Page 23-24, lines 500-504 |
| **Comment 7:** If patients were offered an online survey option, did this bias the recruitment to more technology-capable participants?  Similarly, how did the methods try to avoid recruitment bias for participation capable, available, or willing to participate in research (e.g. were there other research processes that bias for or against certain SES?) | We also offered a paper survey to reduce bias for technology-capable participants. This limitation has also been added in the limitation section.  Additionally, although we offered a paper-based survey option to capture the experiences of patients who may not be technologically savvy, all participants chose the online survey version. This may limit the generalizability of the study findings to less tech-savvy patients. | Page 24, lines 504-506 |
| **Comment 8:** In the **TBQ-15, Dimension 1** would be expected to increase if the patient is adherent to medications, which is desirable. Similar with 4. The other Dimensions, especially Administrative and Financial burden decreasing the burden would be desirable by telehealth. Some, e.g. 5 could go either way. | Thank you for this valuable comment. We have added a correlation matrix table (Table 4) which may answer the relationship between three types of telehealth use and treatment burden. | Page 18 |
| **Comment 9:** Explain why mHealth self-management applications are included together with HCP virtual care applications as they may have different usage and burden expectations. For example, virtual consults from a healthcare provider would be expected to potentially reduce patient travel and other time, but using a mHealth self-management tool would be expected to increase the patient burden, even if it is in a “good” (beneficial requirement) burden time such as helping them take their medications properly or exercising which is their health goal.  Similarly for frequency – HCP virtual care tools would be expected to be only required episodically for visits/consultations, whereas a patient self-management app might be expected to be used daily (in a positive way). These should be differentiated and discussed. | Thank you for your comments. We have teased out different modes of telehealth use and their impact on treatment burden in Table 4.  Additionally, we have adopted a broad definition of Telehealth which includes mHealth since it helped us to provide a comprehensive analysis of different types of telehealth and its impact on treatment burden. The definition of telehealth we adopted was: *the use of technology to provide remote personalized health care to patients.* (22) This broad definition of telehealth includes virtual care, patient portals, telemonitoring devices as well as self-management apps. These technologies enable remote care, facilitating disease monitoring without the constraints of frequent office visits. (21) | Page 18  Page 4 |
| **Comment 10:** The methods would be improved if they evaluated the 5 dimensions of the TBQ-15 as independent or logically grouped associations and if they separated self-management tools in sensitivity analysis. Although, this would likely require a larger sample size to be able to detect significant associations and differences. (already under an ideal sample size to detect significant differences). | Due to the small sample size, we could not conduct a meaningful sensitivity analysis. However, Table 4 may answer some of the reviewer’s comments related to different modes of technology use and their relationship with treatment burden. Moreover, we have also attempted to find the correlation between 5 dimensions of treatment burden and three modes of telehealth use (communication, scheduling and monitoring); however, we found the presented Table 4 was a better representation of our analysis. |  |
| **Results:** | | |
| **Comment 11:** It is unclear what the individual burden scores reflect as only the total score was described out of 150: “On average, participants reported the highest burden in their daily lives due to regular healthcare needs (mean=5.85), followed by financial aspects (mean=5.62), social relationships (mean=5.45), and administrative work (mean=5.43).” Please describe (and the differences seem tiny) | Thanks for this comment. We have added clarification on the scale.  Each dimension was rated on a scale of 0 to 10. | Page 13, line 337 |
| **Comment 12:** Table 2 is very helpful to see the types of use, but as above, they have different ‘burden’ purposes. | We agree, we hope the additional Table 4 will be able to demonstrate the different burden purposes. |  |
| Discussion: |  |  |
| **Comment 13:** As the authors acknowledge, their “hypothesis (H1) anticipated a negative relationship between telehealth utilization and treatment burden, implying that higher use of telehealth would be linked to reduced treatment burden.” And that “This hypothesis was created based on prior literature that suggests telehealth can reduce time, travel, and cost-related burdens.”  Therefore only burdens related to time, travel, and cost would be expected to be lower burden, but if the patient adheres to their treatment recommendations, we would expect medication and other burdens to increase. It is important to know which factors are affected in which direction. | Table 4 suggests that the frequency of telehealth use has a positive relationship with both categories of treatment burden. However, the strength of the relationship varies between the two categories as described in the results section. | Page 18 |
| I don’t think the goal of telehealth is to have patients spend less time (burden) on self-management, rather only on the aspects of care that are not required to improve their health, such as some areas of logistics.  In fact, contrary to the authors interpretation, I would posit that people of lower SES would stand to benefit even greater from certain efficiencies offered by various forms of telehealth, at the same time also benefit from greater ‘burden’ (time and effort) of engagement in self-management of their chronic conditions so they reach their health goals. However, equity of access and appropriateness of ease of use of the technology for the specific populations would be key to achieve those goals.  This perspective should be discussed to balance their argument | Thank you for this great comment and we have added this in the discussion section.  Overall, telehealth can significantly reduce the administrative and financial burdens for patients with chronic conditions. Patients with low SES, in particular, can benefit from the efficiencies offered by telehealth, such as reduced paperwork and travel. However, they may also experience better health outcomes due to higher engagement with health-promoting activities. Ensuring equitable access and making the technology easy to use for these specific populations is crucial to realizing these benefits. | Page 23, line 483-488 |
| **Reviewer 2:** | | |
| **Comment 14:**  One very important piece that this article misses is records of the type of phones people used to access telehealth services. This can affect user experience and ultimately impact on treatment burden. | We agree, that the user interface for each phone can vary widely. We have added that in our limitation section.  Furthermore, our study did not collect information such as the specific technology used by patients as well as patients’ attitudes towards technology use. Both of these factors could potentially impact patients’ experienced treatment burden while using telehealth. | Page 23-24, lines 500-510 |
| **Comment 15:** Secondly, a question about the participants use behaviour of smartphones and computers can impact on this as well. Not everyone like computers or phones regardless of their SES. | We agree, that participants’ perception of phones and computers can impact their experience as well. However, we did not collect this data. We have added the following sentence in the future research recommendation section.  Furthermore, our study did not collect information such as the specific technology used by patients as well as patients’ attitudes towards technology use. Both of these factors could potentially impact patients’ experienced treatment burden while using telehealth. | Page 23-24, lines 500-510 |
